# A scoping review of approaches to evaluating workflow management systems for bioinformatics users

**DOI:** 10.1093/bib/bbag396

**Published:** 2026-07-20

**Authors:** Marisa Loach, Kayleigh Smith, Wendi Bacon

**Affiliations:** School of Life, Health and Chemical Sciences, The Open University, Walton Hall, Milton Keynes, Buckinghamshire, MK7 6AA, United Kingdom; School of Life, Health and Chemical Sciences, The Open University, Walton Hall, Milton Keynes, Buckinghamshire, MK7 6AA, United Kingdom; School of Life, Health and Chemical Sciences, The Open University, Walton Hall, Milton Keynes, Buckinghamshire, MK7 6AA, United Kingdom

**Keywords:** scoping review, scientific workflows, workflow management systems, workflow system, usability, user experience

## Abstract

Workflow Management Systems (WMSs) make it easier to run bioinformatics analyses by combining tools into reusable workflows. However, selecting the right WMS can be challenging, particularly for users from noncomputational backgrounds. Many reviews of WMSs focus on technical details or only consider usability from the perspective of developers. The first objective of this scoping review was to identify the characteristics of bioinformatics WMSs that are most relevant for life scientists. The second objective was to explore how these characteristics have previously been evaluated. The review included 21 papers published since 2018 that evaluated bioinformatics WMSs based on criteria relating to the user experience. Published papers and websites describing 55 currently available WMSs were also included to identify characteristics highlighted by their developers. Twelve themes emerged from these evaluation criteria and WMS characteristics: Basic Computing, Functions, Security, Scalability, Cost/Efficiency, Sustainability, Usability, Learnability, Reproducibility, FAIRness (Findability, Accessibility, Interoperability, Reusability), Flexibility, and Support. Evaluations focusing on the needs of noncomputational users preferred Graphical User Interfaces and platforms that provided plenty of guidance for users. Papers prioritizing the needs of developers instead favoured text-based interfaces and flexible platforms that gave users greater control. In addition to these contrasting views on what was considered a positive characteristic, differences in how criteria were defined and scored meant that evaluations could not be compared between papers and would be impossible for users to repeat on new or updated WMSs. Users do not currently have a clear approach to follow when selecting a WMS.

## Graphical Abstract

**Graphical Abstract:**
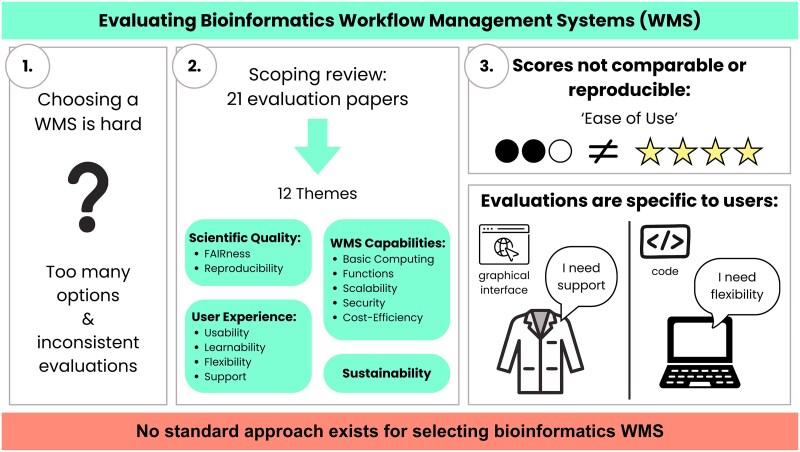

## Introduction

Life scientists face numerous challenges in bioinformatics, many of which can be addressed by selecting the right Workflow Management System (WMS). WMSs are software that enable users to combine tools into reusable workflows, potentially making it easier to find and use tools, to reproduce analyses, and, in some cases, even opening up bioinformatics to those with little or no programming experience. However, WMSs come in many forms, offering different tools, interfaces, and approaches to creating and managing workflows. Users therefore face a new challenge: selecting the most appropriate platform for their analysis.

The choice of WMS will substantially impact the user experience, so users should consider their options carefully before investing time in learning to use a specific system [1]. New bioinformaticians confused by the variety of available tools can choose a WMS that offers a curated selection, where tools may be ready to run without having to manage installation or dependencies [2]. WMSs that maintain up to date tools within their own user-friendly interfaces can address the common issues of usability and software sustainability for bioinformatics tools, which are often designed with little or no user testing and only limited efforts to maintain them [3–5]. Users can work in a single workflow language or interface within a WMS rather than learning and switching between multiple programming languages.

Beyond the experience of interacting with the WMS, researchers can choose platforms that enable them to follow best practices in bioinformatics. A WMS that automatically records details such as the computing environment, tool versions, and parameters can ensure reproducibility. WMSs can also enable researchers to follow the FAIR (Findable, Accessible, Interoperable, and Reusable) guidelines, not just for their data but also for the software and workflows used in analyses [6–8]. A WMS that provides a pre-installed selection of tools can make them more Findable, while one that allows workflows to be exported in the Common Workflow Language (CWL) can make them more Interoperable [9].

Published reviews of WMSs can only help with platform selection if they provide information that is relevant to user needs, and specifically to the needs of users with similar computational backgrounds. Previous systematic reviews have considered usability and platform choice for similar bioinformatics resources such as data repositories [10, 11]. However, little has been done to compare the experience of working with different WMSs from the perspective of an end-user rather than a tool developer. Reviews of WMSs have focused on technical aspects such as computing power and the programming languages used to build them, which aren’t particularly informative for noncomputational users [12, 13]. Where more relevant factors such as usability and reproducibility have been evaluated for WMSs, it has typically been done in a nonsystematic way, with a narrow focus, or using a few subjective criteria as part of a review with a more technical focus. Kouskoumvekaki *et al.* gave their impressions of several WMSs and described how they made bioinformatics more user-friendly, without systematically evaluating them [2]. Cohen-Boulakia *et al.* described characteristics that enabled reproducibility but did not consider other aspects of the user experience [14]. Leipzig rated WMSs for ‘ease of use’ and ‘ease of development’ as part of their discussion of the design philosophies behind different platforms without explaining how they made these apparently subjective judgements [1]. When criteria relating to the user experience are applied more systematically, these evaluations can be hard to find among the technical details or based on the perspectives of software developers who require more power and control [15, 16]. Users cannot easily find the information they need when choosing a WMS.

This review foregrounds the WMS characteristics that users should consider by systematically mapping the relevant criteria from recent reviews. The main objective was to identify the characteristics of bioinformatics WMSs that improve user experience and enable biologists, clinicians, and other users from noncomputational backgrounds to overcome common challenges such as reproducibility. The secondary aim was to examine how these characteristics have previously been evaluated as a step towards helping users make more informed platform choices.

A scoping review approach was chosen since the preliminary literature search suggested the number of studies exploring WMS usability was limited. Inconsistencies in the criteria and evaluation methods also meant there would not be enough comparable outcomes to perform a systematic review [17]. This review covers evaluations of bioinformatics WMSs, which are software that provide access to a selection of bioinformatics tools, pipelines, and customizable workflows.

The review began by gathering recent papers that have evaluated WMSs to determine which criteria have been used to compare these platforms and what types of scoring systems have been applied. The limited and potentially outdated information provided by these papers was supplemented by the characteristics highlighted by the WMS developers themselves in their platform papers and websites. The results reveal the range of characteristics that WMSs can provide to benefit life scientists and the need for a simpler, more objective framework for comparing and choosing a WMS.

## Methods

A scoping review was conducted in line with the Preferred Reporting Items for Systematic reviews and Meta-Analyses (PRISMA) and Joanna Briggs Institute (JBI) guidelines ([17, 18], Supplementary Material). Following identification of the research question, a literature search was performed, and relevant papers were selected against the eligibility criteria as described below and in the published protocol [19].

### Changes from the protocol

The protocol set out to identify papers published up to 5 years before the initial searches were conducted in 2023, but additional searches were performed in 2025 to update the results. The research question was redefined as identifying ‘characteristics’ of WMSs rather than ‘features’, as it emerged during the review process that ‘features’ was used in computational fields to refer to functions of the software rather than the general characteristics that were being reviewed. The definition of a WMS was also refined during the scoping review to distinguish between WMSs and platforms that provide access to bioinformatics tools without allowing workflows to be built and reused. The exclusion criteria were edited to clarify that included papers should methodically evaluate WMSs against specific criteria, rather than simply include an author’s opinions on specific platforms. These changes were made to ensure consistency in the paper selection process and to maintain focus on the research question.

### Search strategy

Searches were conducted in the PubMed, Scopus, IEEE Xplore, and BioRxiv/MedRxiv databases to identify relevant papers that review, compare, or evaluate WMSs. Results were limited to papers published since 2018. The initial search was performed on 6 March 2023, and updates were performed on 3 February 2025.

Searches combined bioinformatics with terms referring to WMSs and keywords representing the concepts under investigation or the types of papers required. Search terms were restricted to titles (or titles and abstracts) to focus on papers where these are the main topics. The full search terms used in the PubMed database are shown in Supplementary Text S1.

The references of included papers were checked for additional papers that met the inclusion criteria. Papers citing the included studies were also identified and checked against the eligibility criteria.

Names of WMSs mentioned in the papers were logged during the selection and extraction phases. Online searches were performed to check which of these WMSs were currently available. The most recent peer-reviewed articles published by the owners/developers of each platform were then identified. Grey literature searches were also performed to identify the official websites or documentation. These additional sources were included as they might describe WMS characteristics that were not evaluated by the review papers.

### Selection strategy

Following deduplication, titles and abstracts were first checked for eligibility against the criteria presented below. Potentially relevant papers were then checked in full to ensure their eligibility and relevance to the research question. Reasons for exclusion were noted during this second check. Papers describing individual WMSs were tagged, and the most recent versions were included as platform papers if they met the eligibility criteria below. A second reviewer independently checked 10% of the results at each stage. Any discrepancies were resolved by a third party.

### Eligibility criteria

Peer reviewed articles, preprints, and reviews published in English since 2018 were included if they compared, benchmarked, or evaluated bioinformatics WMSs against specified criteria. Papers were not included if they only expressed opinions on WMSs within their text in order to limit the results to those most relevant to the objective of exploring how WMSs have previously been evaluated. WMSs were defined as platforms, workbenches, or toolkits that enable the use and management of a variety of tools, workflows, or pipelines. Platforms that did not allow creation or reuse of workflows were not considered WMSs. WMSs were restricted to general or specialized platforms that allowed analysis of sequencing data (e.g. genomics, transcriptomics) to ensure comparability and limit the search. Platforms focusing on other domains such as image-based analyses were therefore excluded. Papers published before 2018 (5 years before the initial search) were excluded as the aim was to identify the current characteristics of WMSs.

Named WMSs were identified during the selection and extraction phases. The most recent peer-reviewed papers presenting the currently available WMSs and written by the owners/developers were included even if they did not appear in the search results or were published before 2018. WMSs were considered available if there were active links for using or installing them, without checking whether they were currently being maintained. Papers that focused on individual tools, workflows, or instances of a WMS were excluded. The official websites and online documentation for each platform were used to complete the data extraction where items were missing or outdated in the peer-reviewed literature. Online material from sources other than the WMS owners/developers was not consulted.

### Data extraction

Summaries of the included sources and relevant data items were extracted by a single reviewer into a form with defined fields. Details of each source including full citations for journal articles, URLs, and access dates for online material were recorded. The criteria used to compare or evaluate WMSs in terms of the user experience were extracted from the included review papers, along with their definitions and details of any scoring system used to evaluate the platforms. Characteristics highlighted by the platform creators in section headings, boxes, or lists of key features as benefitting users were extracted from the platform papers and websites, along with summaries of their descriptions. Basic information about the WMSs, including their costs and interface types, was also recorded. Different extraction forms were used for review papers and platform papers or websites (Supplementary Table S1).

Both extraction forms were initially tested against one paper or platform. After the forms were refined and definitions were created for each item, a second reviewer independently performed the same extractions to check the accuracy and completeness of the first reviewer. One additional item, the target user or audience, was identified as relevant during the extraction process and added to the form.

In the absence of a suitable standard for critical appraisal of evaluation papers, particularly given the aim of mapping the heterogeneity of criteria and scoring systems rather than comparing the outcomes, this optional step in the PRISMA and JBI guidelines for scoping reviews was not performed ([17, 18], Supplementary Material). However, the eligibility criteria ensured methodological comparability between the included papers by requiring them to evaluate bioinformatics WMSs against specified criteria and a simple descriptive appraisal of each study was performed based on extractions of the aims, criteria definitions, and scoring systems.

### Data synthesis

The criteria and scoring systems used by different papers to evaluate WMSs were summarized in tables. Basic information about each WMS obtained from platform papers and websites was collated into a table. The number of platforms using each mode of access and interaction were counted to provide an overview of available types of WMSs. An inductive approach was taken for classifying the criteria used to evaluate WMSs by theme, based on similarities in their names, definitions, and any additional descriptions of their meaning and significance for users. Given the heterogeneity of the evaluation criteria and the lack of domain-specific standards for classification, analytical themes were identified without enforcing mutually exclusive categories. Some overlap between themes was allowed to reflect the interrelatedness of usability-related concepts. Where the original papers distinguished between related concepts, such as ease of learning and ease of use, these distinctions were preserved to achieve the aim of mapping existing criteria. Characteristics that were not relevant to the user experience (e.g. technical details of the WMS) were not categorized as they were not within the scope of this review. The themes that emerged from the evaluation papers were used to classify the characteristics highlighted by the platform developers. New themes were defined at this stage if the WMS materials covered topics not considered by the evaluation papers.

## Results

**Figure 1:**
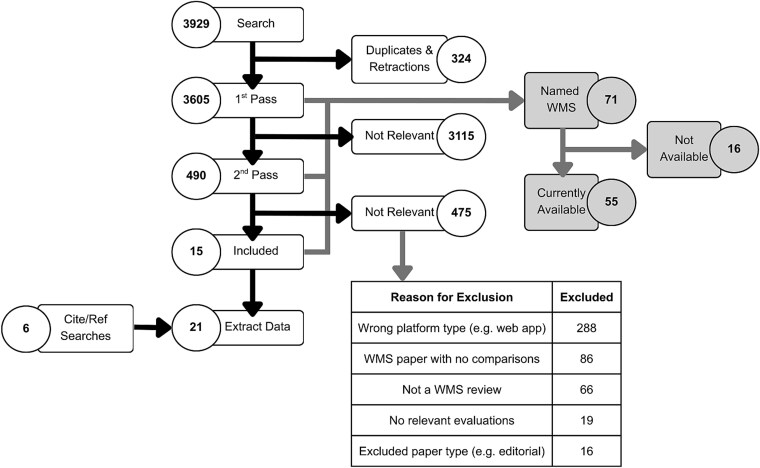
Flow chart of review process. Numbers of papers found, included and excluded at each stage of the search and selection process. The numbers of named and available WMSs identified throughout the process are also shown.

The search process identified 3605 papers, following deduplication, of which 15 met the inclusion criteria (Fig. 1). Six additional papers were identified through the reference and citation searches, making a total of 21 included papers (Table 1). Papers describing individual WMSs were tagged and the names of any WMSs mentioned or evaluated were recorded. The platform papers and websites were checked to identify 55 currently available WMSs.

**Table 1:** Included papers.

**Paper title**	**Author (date)**	**Summary (basic aim/method)**	**Scope**	**Criteria defined**	**Scored**	**Individual/group evaluations**
**Design considerations for workflow management systems use in production genomics research and the clinic**	Ahmed *et al.* 2021 [15]	Technical test of workflow languages/WMS by running two pipelines in multiple computing environments.	Bioinformatics	Yes	No	Individual
**CSI NGS Portal: An Online Platform for Automated NGS Data Analysis and Sharing** ^**a**^	An *et al.* 2020 [20]	Comparison of CSI NGS to other platforms.	NGS data	Named but not defined	Yes	Individual
**Challenges for the development of automated RNA-seq analyses pipelines**	Beukers and Allmer (2023) [21]	Compares WMSs for creating automated RNA-seq pipelines.	RNA-Seq	Named but not defined	Yes	Individual
**On distributed collaboration for biomedical sciences**	Boujdad *et al.* (2019) [22]	Describes options for cloud computing and compares platforms.	Biomedical	Some	Yes	Individual
**Anduril 2: upgraded large-scale data integration framework** ^**a**^	Cervera *et al.* (2019) [23]	Supplementary material includes table comparing Anduril to other platforms.	Bioinformatics	Named but not defined	Yes	Individual
**Constructing lightweight and flexible pipelines using Plugin-Based Microbiome Analysis (PluMA)** ^**a**^	Cickovski and Narasimhan (2018*)* [24]	Compares types of WMSs and discusses user needs while presenting the PluMA platform for microbiome analysis.	Microbiome	Yes	Yes	Grouped
**Developing and reusing bioinformatics data analysis pipelines using scientific workflow systems**	Djaffardjy *et al.* (2023) [25]	Discusses the challenges for pipeline development and how WMSs address them. Also determines how often public workflows were reused on different WMSs.	Bioinformatics	Yes	No	Individual
**System for Quality-Assured Data Analysis: Flexible, reproducible scientific workflows** ^**a**^	Fowler *et al.* (2019*)* [26]	Describes SyQADA, a system for increasing workflow reproducibility, and compares types of platforms.	Bioinformatics	Named but not defined	Yes	Individual
**Using prototyping to choose a bioinformatics workflow management system**	Jackson *et al.* (2021) [16]	Suggests a prototyping strategy for developers to select which WMS to build tools for.	Bioinformatics	Yes	Yes	Individual
**uap: reproducible and robust HTS data analysis** ^**a**^	Kämpf *et al.* (2019) [27]	Describes uap and compares it with other WMSs.	NGS data	Yes	Yes	Individual
**Improving data workflow systems with cloud services and use of open data for bioinformatics research**	Karim *et al.* (2018) [28]	Discusses use of cloud computing and open data in bioinformatics, including criteria that WMSs need to meet, based on a systematic review.	Biomedical	Yes	Yes	Individual
**Maser: one-stop platform for NGS big data from analysis to visualisation** ^**a**^	Kinjo *et al.* (2018) [29]	Describes Maser and compares it to other WMSs.	NGS data	Named but not defined	Yes	Individual
**Criteria for the Evaluation of Workflow Management Systems for Scientific Data Analysis**	Kiran *et al.* (2023) [30]	Scoping review of papers comparing WMSs to select consensus criteria for evaluations.	Bioinformatics and beyond	Yes	Yes	Individual
**Watchdog 2.0: New developments for reusability, reproducibility, and workflow execution** ^**a**^	Kluge *et al.* (2020) [31]	Describes Watchdog and compares it to other WMSs.	Bioinformatics	Named but not defined	Yes	Individual
**Evaluating Workflow Management Systems: A Bioinformatics Use Case**	Larsonneur *et al.* (2018) [32]	Evaluates efficiency of WMSs.	Bioinformatics	Named but not defined	Yes	Individual
**Comparative analysis of workflow platform in support of in silico oncology**	Marinova and Lazarov (2019) [33]	Evaluates types of WMSs used in oncology.	Oncology	Yes	Yes	Grouped
**BioSAILs: versatile workflow management for high-throughput data analysis** ^**a**^	Rowe *et al.* (2019) [34]	Describes BioSAILS and compares it to other WMSs.	NGS data	Named but not defined	Yes	Individual
**Computational Strategies for Scalable Genomics Analysis**	Shi and Wang (2019) [35]	Review of cloud computing and hardware solutions for big genomics data, including WMSs.	Genomics	Named but not defined	Yes	Grouped
**TOGGLe, a flexible framework for easily building complex workflows and performing robust large-scale NGS analyses** ^**a**^	Tranchant-Dubreuil *et al.* (2018) [36]	Describes TOGGLe and compares it to other WMSs.	NGS data	Named but not defined	Yes	Individual
**Managing Complex Workflows in Bioinformatics: An Interactive Toolkit With GPU** ^**a**^ **Acceleration**	Welivita et *al.* (2018) [37]	Describes BioWorkflow and compares it to other WMSs.	Bioinformatics	Yes	No	Individual
**Reproducible, scalable, and shareable analysis pipelines with bioinformatics workflow managers.**	Wratten (2021) [38]	Evaluates WMSs.	Bioinformatics	Yes	Yes	Individual

Summary of papers selected for inclusion. Papers are categorized based on whether their criteria were defined, scores were assigned, and evaluations performed on individual WMS or groups of similar platforms. NGS, Next-Generation Sequencing.

^a^Indicates evaluation papers that were also platform papers.

The included papers varied in the clarity and completeness with which they defined and scored their evaluation criteria (Table 1). Seven papers explicitly defined criteria or desirable features and then scored WMSs based on these. Another 11 papers did not fully define their criteria, limiting the transparency and comparability of their evaluations, but did score WMSs based on named characteristics. Three studies (Ahmed *et al.*, Djaffardjy *et al.*, and Welivita *et al.*) named or described the criteria a WMS should meet and discussed which WMSs met them but did not have a set scoring method, limiting the reproducibility of their approaches [15, 25, 37].

Some papers focused on specific aspects of WMS use, such as Boujdad *et al.* on cloud computing or Shi and Wang on scalability [22, 35]. Ten papers were written by developers of specific platforms but were included as they also provided broader evaluations of WMSs against named criteria [20, 23, 24, 26, 27, 29, 31, 34, 36, 37]. For these papers, evaluation criteria were extracted once and analysed, along with the evaluation papers, while any additional platform-specific characteristics highlighted by the developers were included with the other WMS materials, ensuring that each element was only included in one part of the analysis. The remaining papers covered a range of criteria and platforms, typically across broad domains such as bioinformatics or biomedical analysis.

**Figure 2:**
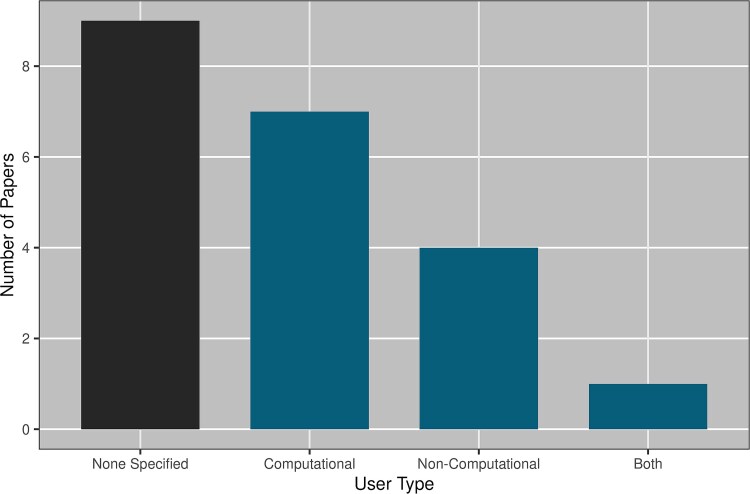
Types of users. User types identified in the included papers as the focus of their evaluations, showing that most papers did not specify users and noncomputational users were considered less often than computational users. Computational users include papers focused on more technical aspects of WMSs, developers, or users with programming experience. Both indicates a paper targeting both computational and noncomputational users simultaneously.

The papers also differed in the types of users whose needs were considered, which could shape the characteristics that were favoured by the reviewers. Most papers did not specify a user type, but noncomputational users were less commonly considered by those that did (Fig. 2). Two of these studies (Ahmed *et al.* and Larsonneur *et al.*) focused on technical aspects of WMSs but did include some criteria relevant to computational end-users [15, 32]. Of the papers which discussed specific types of users, three (Cickovski and Narasimhan, Jackson *et al.*, and Shi and Wang) focused on tool or algorithm developers, which could reduce their relevance for noncomputational users [16, 24, 35]. Seven papers focused on end-users with different levels of computational experience. Two papers, Fowler *et al.* and Cervera *et al.*, discussed the needs of users with some programming experience [23, 26] and four (Beukers and Allmer, Kinjo *et al.*, Kiran *et al.*, and Tranchant-Dubreuil *et al.*) based their evaluations around the needs of users without computational experience [21, 29, 30, 36], while Wratten *et al.* considered the needs of both types of users [38].

### Evaluated platforms

**Figure 3:**
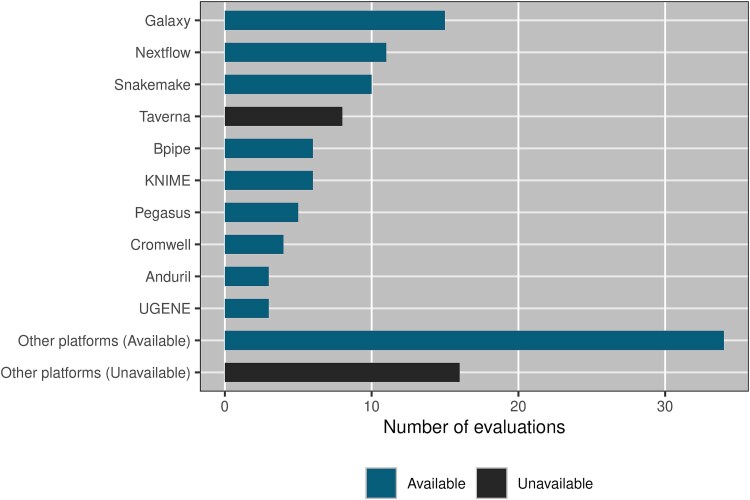
Most frequently evaluated platforms. The most frequently evaluated WMSs, showing that a small number of platforms are commonly evaluated and some evaluated WMSs are no longer available. Bars indicate the number of times each WMS was evaluated by the included papers. The top 10 most-often evaluated WMSs are shown. Other bars show the totals for the 25 available and 12 unavailable WMSs that were evaluated only once or twice. Grey bars indicate platforms that are no longer available. Non-WMSs evaluated by the papers are not included in this figure.

A total of 55 currently available (although not necessarily maintained) WMSs were identified during the review (Fig. 1). The included papers evaluated 34 of these WMSs, as well as 13 platforms that were no longer available when this review was performed. The 21 other available WMSs were identified during the search and extraction process as they were named but not evaluated, along with three more unavailable platforms. Even though the search was restricted to papers published after 2018, 16 of the 71 identified WMSs had already ceased to function, revealing how quickly the literature can become outdated and reducing the relevance of evaluation papers for users currently choosing a WMS (Fig. 3).

Only platforms meeting the WMS definition set out in the Eligibility Criteria were included. Many of the named and evaluated platforms were web apps that allowed users to run data through various tools but did not meet the requirement of allowing users to create workflows (e.g. GeneCloudOmics, GeneTrail, OMnalysis [39–41]). When the searches were updated, an increase in the number of Artificial Intelligence (AI) and chat-based tools was apparent, but most were intended for exploratory analysis rather than workflow creation (e.g. RiboChat, GeneGPT, BioMaster [42–44].

Some WMSs were frequently selected for evaluation, with Galaxy, Nextflow, Snakemake, and the now unavailable Taverna appearing most often, while many others were only evaluated once or twice (Fig. 3). Other types of tools, pipelines, and applications, as well as workflow languages (e.g. CWL, WDL) and engines (e.g. Toil), were sometimes evaluated alongside the WMSs.

### Types of Workflow Management System

The platform websites and latest publications from the developers of the 55 currently available WMSs were identified. Basic information about the platforms and the characteristics highlighted by their creators were extracted (Supplementary Table S2).

**Figure 4:**
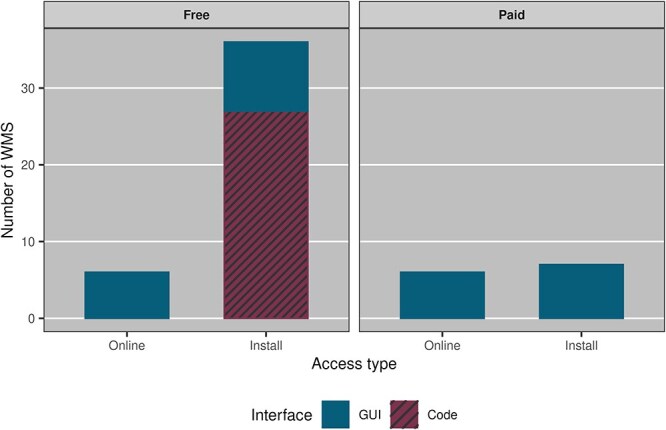
WMS types. Distribution of the 55 available WMSs by cost, access method, and interface type. Systems are grouped by cost (free or paid), with bars showing access type (online or installed). Solid bars are graphical user interfaces (GUI) while striped bars are code-based. Code-based WMSs were only observed among free, installable platforms. WMSs that offered multiple cost, access, or interface options are grouped according to the most prominent option in their materials.

Currently available WMSs varied in terms of the cost of using them, whether software or packages needed to be installed, and how users interacted with their workflows (Fig. 4). Most WMSs were free to use, although some restricted usage or offered additional paid services, but a smaller number of fully commercial platforms were also available. While GUI-based platforms were evenly distributed across both cost and access types, all the code-based WMSs were primarily free platforms that required installation. Users had options ranging from simple command line tools for running script-based workflows using their own computing resources to cloud-based graphical interfaces with drag-and-drop workflows (Supplementary Table S2). Some WMSs offered users a choice between GUI or code-based workflows, or between online and installed platforms, increasing the options for users and the complexity of evaluating platforms.

The large number of available WMSs would make it impractical for any one review to compare all platforms, and, as seen for those reviews that included WMSs that are no longer available, the results can quickly become outdated (Fig. 3). The wide range of WMS types and the different types of users who might be interested in the results are also likely to make it harder to select evaluation criteria that can compare all platforms in a manner that assists all users in choosing a WMS.

### Evaluation criteria

The criteria in the included papers ranged from technical metrics to evaluate performance through to subjective assessments of how easy the authors found the platforms to use. The full list of criteria relating to the user experience of WMSs, together with any definitions provided in the original papers, is provided in Supplementary Table S3.

Most of the included papers did not explain how or why they had chosen their criteria. Jackson *et al.* made it clear that their criteria were based on their own needs while developing a specific tool, which is why their criteria include the required and useful functions for their Riboviz workflow [16]. Marinova and Lazarov copied the criteria defined by Lepizig, a paper published before the search period for this review that did not justify its own choice of criteria [1, 33]. Fowler *et al.* chose criteria they considered to be key features of specific platforms (e.g. bpipe) [26]. Kiran *et al.* based their criteria on the consensus score calculated by identifying the criteria used in a selection of previous reviews (including Beukers and Allmer, Jackson *et al.*, Karim *et al.*, Larsonneur *et al.*, and Wratten *et al.*) [16, 21, 28, 30, 32, 38]. However, the authors didn’t always follow their method as they included security as a critical criterion despite its low consensus score.

Ten papers simply named their criteria without defining them, making it harder to understand exactly what the authors were evaluating, to identify comparable criteria across papers, or to classify them by theme. The definitions provided by the 11 other papers ranged from brief descriptions to extensive discussions in the main text (Supplementary Table S3). Some specified clear requirements or metrics, such as the number of citations found for each platform in PubMed (Beukers and Allmer; Kiran *et al.*) [21, 30]. Other criteria were more subjective, such as Jackson *et al.*’s evaluation of the supporting documentation based on its readability and utility [16]. Some definitions were entirely dependent on the previous experience of the evaluators, such as Cickovski and Narasimhan’s ‘Language Flexibility’ criterion, which was met if they were able to develop in their preferred language on the platform [24].

Even when similar-sounding criteria were used in multiple papers, there were often differences in how those criteria were defined. Three papers listed ‘Ease of Use’ among their criteria, but this was defined as a WMS that enabled use with little or no training overheads by Karim *et al.*, as the effort required to use or share workflows by Marinova and Lazarov, and as the type of computing environment by Wratten *et al.*, who gave their highest scores for this criterion to WMSs that provided a GUI [28, 33, 38].

The characteristics highlighted by the platform developers were not as narrowly defined as the criteria used in the evaluation papers, so it wasn’t always possible to assign a single theme. Multiple themes were assigned to the broader characteristics that covered different aspects of the user experience. The names, descriptions, and themes assigned to each highlighted characteristic are available in Supplementary Table S4.

### Themes

Twelve common themes emerged from the criteria used to evaluate WMSs and the characteristics highlighted by WMS developers (Table 2). Several themes covered requirements for ensuring the WMSs could be used for particular purposes, such as the Basic Computing requirements for installation or the availability of specific Functions. Security measures, such as password protection, and the Scalability of the platforms were also mentioned. Sustainability was another common theme in the evaluation papers, with WMSs that had been around for longer, were currently maintained, and had active user communities being considered more dependable. Communities were evaluated as signs of popularity and stability, rather than for the support they can provide to users.

**Table 2:** Themes.

**Theme**	**Definition**
**1. Basic Computing**	Requirements for installing and running the WMS (e.g. environments it can be run in)
**2. Functions**	Availability of the tools, data types, or workflow functions needed to perform the desired analyses. **Common subthemes:** **Scope:** General scope of the WMS—a quick insight into the types of analyses it will be possible to perform, without guaranteeing specific tools or functions will be available **Data:** Available data types for inputs/outputs, availability of conversion tools, upload from directories, or local storage **Data Management:** Functions for renaming, moving, storing, or otherwise managing data **Tools:** Available tools or types of tools **Workflows:** Functions for building, editing, or running workflows, including modularity (the ability to take out and recombine parts of a workflow) and progress checking (the ability to monitor running workflows) **Collaboration:** Functions that allow multiple users to work together on the same analysis or workflow (NOT on separate copies of a data/workflow that has been shared)
**3. Security**	Measures that keep data, analyses and workflows private, or allow secure access/sharing, including compliance with specific standards
**4. Scalability**	The size of data or extent of the computational demands that the WMS can handle
**5. Sustainability**	How likely the WMS is to continue to be available in a usable state **Common subthemes:** **Established:** Stability of the WMS—how it has been around for **Maintained:** Current state of the WMS—whether it is being actively updated and maintained **Community:** How likely the WMS is to continue being used and maintained—size or activity of the user/developer community **Popularity:** How often the WMS is used (generally or in a specific field)—the number of users or citations
**6. Usability**	The experience of using the WMS **Common subthemes:** **Install:** How easily the WMS can be set up, installed, or used for the first time **Interface:** How users interact with the WMS (e.g. through a GUI) **Development:** Ease of developing tools using the WMS **Use:** Ease of using the WMS to create workflows and run analyses **Robustness & Error Handling:** Features for preventing, identifying, or tackling errors **Accessibility:** Features that make the WMS usable for all
**7. Learnability**	How easily users can learn to use the WMS or to perform bioinformatic analyses through it (e.g. availability of learning materials)
**8. FAIRness**	Whether data, workflows, and the WMS itself adhere to the FAIR principles. **Covers:** **Findability,** e.g. search functions for tools or shared workflows. **Access,** e.g. whether the platform is open access **Interoperability,** e.g. import/export workflows in formats that can be used in different computing environments or other WMSs **Reusability,** e.g. ability to share and reuse workflows
**9. Reproducibility**	Whether the WMS enables analyses to be repeated, replicated, or reproduced **Common subthemes:** **Provenance:** Automatic recording of inputs, tools, versions, parameters etc **Transparency:** Visibility of the commands and code used
**10. Cost/Efficiency**	How much it costs to run an analysis, in terms of time and/or money
**11. Flexibility**	How much control the user has to customize the WMS and express any workflow pattern or function within it (often with limited guidance as analyses are more custom-built)
**12. Support**	How much guidance (e.g. support services, suggested values) is provided to help users through their analysis (which could limit their control or restrict options)

Definitions of the 12 themes that emerged from the evaluation criteria used by included papers and the characteristics highlighted by WMS developers. Common sub-themes are described for themes that included recurring topics.

Other themes related to the experience of using the WMSs and the common challenges they helped users to address. Usability was considered in terms of the ease of installation, ease of use, and ease of development, as well as the type of interface and how errors were handled. Learnability covered the availability of documentation and training materials. Many evaluations considered how well the WMSs enabled Reproducibility and FAIRness of data and workflows, through characteristics such as provenance tracking and workflow sharing. Two themes were defined in opposition to each other, with Flexibility focusing on the ability of the user to take control or adapt the WMSs to their preferences, while Support focused on how well users were guided through their analysis.

Certain themes aligned with established software quality and user-interaction frameworks, while reflecting the blurred boundaries between different aspects of user experience, both in these standards and in the included papers [45, 46]. Themes relating to the capabilities of WMSs (Basic Computing, Functions, Security, Cost/Efficiency, and Scalability) broadly corresponded to characteristics defined in ISO 25010, particularly functional suitability, security, and aspects of performance efficiency [45]. Themes relating to user experience (Usability, Learnability, Flexibility, and Support) broadly aligned with aspects of the usability and interaction principles described in ISO 9241-110, including conformity with user expectations, error robustness, learnability, controllability, and self-descriptiveness [46]. However, these usability-related themes are inherently interrelated, and clear boundaries are not consistently defined between them in either standards or the included papers. The themes were defined to preserve distinctions in the evaluation papers, such as the use of separate criteria for learning materials and ease of use [16, 30, 38]. The inductive approach also identified the domain-specific themes of Reproducibility and FAIRness in WMS evaluations, which are not covered by general software standards but are central to the evaluation of bioinformatics WMSs.

The themes were broadly consistent across the criteria used in independent and platform-associated evaluation papers (Supplementary Table S5a). Differences in the number of criteria assigned to certain themes reflected variation in study size rather than systematic differences between these paper types. Variations in how themes were applied within the 21 evaluation papers were more closely tied to the user types they considered, although for the developer-authored papers, this likely reflects the target users of their WMSs.

Some differences were apparent between the evaluation papers and the additional WMS materials. The themes that emerged from the evaluation papers broadly encompassed the characteristics highlighted by developers, suggesting that both sources address similar concerns for users. However, the characteristics highlighted by platform developers tended to reflect the specific strengths and features of their systems (e.g. GUI-based Closha emphasizes its user-friendly interface, while the code-based Nextflow highlights other aspects of Usability such as error handling) [47, 48]. While it is possible some of the additional characteristics identified in the WMS materials were in fact evaluated by papers that didn’t fully define their criteria, they may reflect differences in the aims or perspectives of the reviewers and developers. One theme, Cost/Efficiency, was only discussed by some of the commercial WMS developers but not considered by any evaluation papers. Wratten *et al.* did mention that cost and time-to-result were becoming more important due to increasing use of cloud services but did not include this in their criteria [38]. Online or cloud-based WMSs also highlighted Functions that enabled collaboration, which were not explicitly evaluated by any papers, perhaps because these are newer or less commonly available features that have not yet been included in evaluation criteria (Supplementary Figs S1 and S2). The accessibility component of Usability was only mentioned by one platform, Galaxy, suggesting it is not commonly considered by either reviewers or WMS developers, despite its potential impact on users [49]. Inclusion of the WMS materials enabled these emerging features and priorities to be captured in this review.

**Figure 5:**
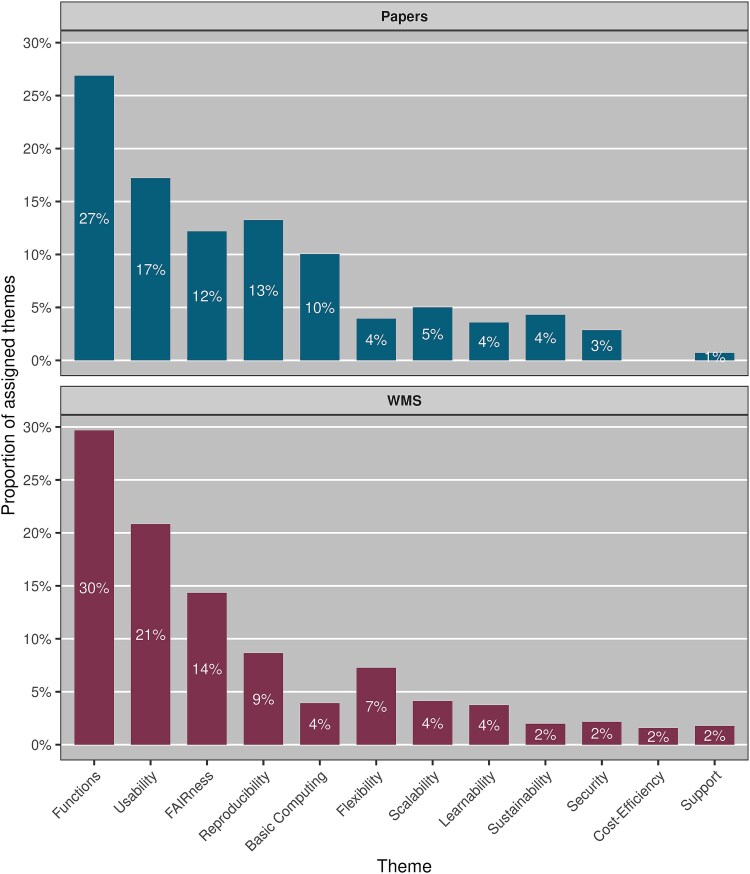
Themes assigned to evaluation criteria and WMS characteristics. Top chart shows the proportion of criteria assigned to each theme. Each criterion was assigned to a single theme that most closely matched its definition. Bottom chart shows the proportion of times each theme was assigned to the characteristics highlighted by WMS developers. Some characteristics were assigned to multiple themes as they were more broadly defined. Values on bars are rounded to the nearest percent.

The frequency at which the themes appeared in previous evaluations and WMS materials shows how often this information is available for users to consider when selecting a platform (Fig. 5). The frequency of the subthemes is shown in Supplementary Figs S1 and S2. Functions, such as the availability of certain tools, data types, or workflow patterns, were discussed most often. Usability, FAIRness, and Reproducibility were also frequently evaluated or described by WMS developers. However, while it seems that these themes are consistently considered important, their frequency does not necessarily reflect their importance for the reviewers, WMS developers, or the users whose needs are ultimately being considered. Some sources focused on specific aspects of the user experience, such as Reproducibility in Fowler *et al.* or Usability in Marinova and Lazarov, increasing the occurrences of those themes [26, 33]. Sources that broke their criteria down into many separate points also increased the frequency of certain themes, without necessarily considering them more important than those that grouped their ideas into a few broad points. Beukers and Allmer listed 35 criteria detailing specific tools and other functions they believed were important for RNA-seq analysis, while Jackson *et al.* used just two criteria to assess whether the necessary and important functions for developing their tool were available [16, 21]. Themes that appeared less often, such as Cost/Efficiency and Learnability, could therefore still be important to users.

**Figure 6:**
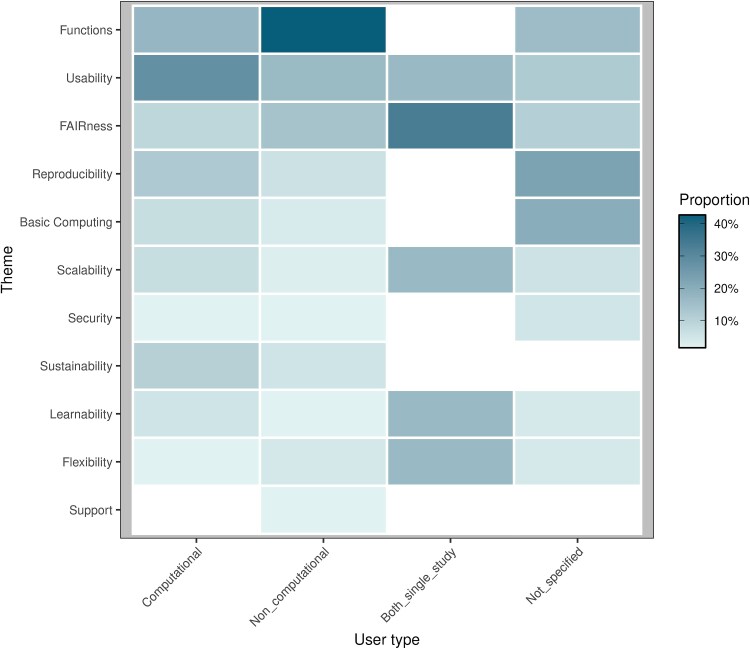
Heatmap of themes by user type. Distribution of evaluation themes across papers focusing on different user types. Values represent the proportion of criteria assigned to each theme within the user category. Computational users include papers focused on more technical aspects of WMSs, developers, and users with programming experience. The Both category represents a single study, Wratten *et al.* [38].

The main themes appear to be relevant to all types of users as, with the exception of Support, they were evaluated by papers focusing on the needs of both computational and noncomputational users (Fig. 6, Supplementary Table S5b). The apparent differences between these two groups are largely due to the influence of a single paper, Beukers and Allmer, which included a high proportion of criteria relating to Functions and was the only paper to consider Support [21]. Only one paper, Wratten *et al.*, attempted to evaluate WMSs for both computational and noncomputational users simultaneously, limiting the themes covered by this user category [38].

Despite the overall similarities, a closer look at how criteria were described reveals differences in which aspects of these themes were considered important (Supplementary Table S3). The Functions required by papers focusing on noncomputational users were more likely to be specific tools or ready to use pipelines [21, 38]. Papers focusing on developers or users with programming experience were more interested in workflow functions that allowed tools to be combined and run in different ways, such as the ability to create loops, nested workflows, or conditional steps [15, 16, 26]. The workflow functions included in the papers focused on noncomputational users related to scheduling or automating workflow runs, rather than creating complex structures [21, 30]. Both Beukers and Allmer and Kiran *et al.* did also include ‘workflow flexibility’ among their criteria, but for them, this appeared to represent a trade-off between expressiveness and human readability, rather than a preference for workflow complexity.

The different needs of computational and noncomputational users were particularly apparent when it came to how criteria relating to WMS interfaces were scored within the Usability theme. Papers focused on developers, such as Jackson *et al.*, considered a text-based interface to be more usable while those considering noncomputational users, such as Kiran *et al.*, preferred a GUI [16, 30]. Wratten *et al.* used two criteria that were scored in opposite directions, perhaps reflecting their consideration of the needs of both types of users [38]. Usability (as ‘Ease of Use’) was scored highest for GUIs, while these interfaces were given the lowest scores for Flexibility (‘Expressiveness’).

The main themes appeared consistently across the papers and websites associated with a variety of different WMSs, including both free and paid platforms, as well as WMSs with text-based and graphical interfaces (Supplementary Table S6). However, differences in target users may explain why certain themes were more common in certain WMS materials. Learnability and Support were often discussed by the developers of GUI-based WMSs, suggesting a stronger focus on the needs of new users and those from noncomputational backgrounds since these platforms are often designed to be suitable for users without programming skills.

### Scoring systems

Identifying which WMS characteristics matter for life scientists isn’t enough to ensure these users can select the right platform. The secondary objective of this review was to explore how these characteristics have been evaluated. The scoring systems used in previous reviews not only explain how value was assigned to the WMS characteristics discussed above but also determine whether the results are comparable or reproducible.

Most (18 of the 21) included papers explicitly assigned scores against their chosen criteria, although three of these papers evaluated groups of similar platforms rather than individual WMSs (Table 3). Eleven papers simply scored on whether the criteria were met or if a specific feature was present [16, 20, 22–24, 26, 28, 29, 31, 32, 34]. Six others used multiple levels (between three and five) to further differentiate between platforms [21, 27, 33, 35, 36, 38]. Additional information was sometimes included to elaborate on these scores and some papers allowed for extra options, such as ‘Depends’ or question marks when the outcome was unclear. Karim *et al.* was the only paper that gave an overall score for each platform and type of platform by summing up the total number of criteria that were met [28]. The final paper, Kiran *et al.*, took a different approach, defining numbered levels for each of their criteria and then listing which levels each platform achieved [30]. Some platforms achieved multiple levels and were assigned multiple numbers for those criteria.

**Table 3:** Scoring systems used to evaluate WMSs.

**Review paper**	**Scoring system**	**Example**
An *et al.* [20]	Individual platforms given mostly Yes or No, but with additional options (e.g. Limited, In progress) or categories (e.g. Dynamic or Static data visualization) used in some cases. Number of NGS pipelines was specified.	Yes
Beukers and Allmer [21]	Individual platforms were scored with short text summaries for each criterion and highlighted using a ‘traffic light’ system of Red, Yellow, and Green where Green indicated ‘good’ (colours not shown here).	Description of what the platform offers. Some limitations.
Boujdad *et al.* [22]	Individual platforms were given an ‘X’ for criteria they satisfied to some extent Architecture types were scored as low (−), medium (+), or high (++) for scalability and security and as straightforward (+) or not straightforward (−) for reproducibility	X
Cickovski and Narasimhan [24]	Groups of similar platforms scored Yes, No, or Depends	Yes
Cervera *et al.* [23]	Individual platforms given ✓ or ×, with short text or numbers used in some cases (e.g. number of years of development, workflow loops described as ‘not nested’)	X
Fowler *et al.* [26]	Individual platforms given Yes, No, ‘?’, or a variety of one-word evaluations (e.g. Under test, almost as-is, Detailed)	No
Jackson *et al.* [16]	Individual platforms scored as Yes/No with some additional text used in table or footnotes to explain scores (e.g. ‘Containers only’, ‘The lack of support for conditional exclusion is a restriction of CWL, not Toil’)	No
Kämpf *et al.* [27]	Individual platforms scored as empty, half-filled, or filled circles, representing not met, partially met, and fully met A − was used when information was not available in the papers, documentation, or manuals for the WMS	●
Karim *et al.* [28]	Individual platforms scored as 1 or blank. Characteristics scored as 1 were higlighted in green (colour not shown here). Totals given for each characteristic (across platforms) and each platform, within the three categories of criteria (IT characteristics, Human interface, Public resources) and as overall totals	1
Kinjo *et al.* [29]	Individual platforms given ✓ or left blank, text used for some criteria (e.g. whether ‘single’ or ‘multiple’ workspaces available).	✓
Kiran *et al.* [30]	Individual platforms were evaluated. Numbers were assigned based on the level at which the platform met each criterion. Multiple numbers were assigned in some cases where the definitions for the levels weren’t cumulative. Additional annotations were sometimes used to explain scores (e.g. ‘depends on the developer’) and one ‘?’ score where level was unclear. Status was given as open or closed source, commercial, or freely available. The number of citations in Pubmed was given.	1, 3, 4
Kluge *et al.* [31]	Individual platforms scored Yes or No, with short text used in some cases (e.g. name of area where tools and workflows can be shared, names of containers used for deployment)	No
Larsonneur *et al.* [32]	Individual WMSs scored Yes or No, with additional text used to provide additional information (e.g. ‘Yes (optional)’) Platforms were ranked from 1 to 5 for technical criteria (beyond the scope of this review) and global rank calculated	Yes (optional)
Marinova and Lazarov [33]	Groups of similar WMSs scored 1–5 stars Criteria and scores taken from Leipzig, but without using the additional half-star levels from the original	★★★
Rowe *et al.* [34]	Individual platforms scored Yes or No, with occasional alternatives (e.g. ‘Pending’, ‘Somewhat’)	Yes
Shi and Wang [35]	Groups of similar WMSs given 1–3 ‘+’ signs	++
Tranchant-Dubreuil *et al.*	Individual platforms given a − or 1–3 + signs, number of integrated tools given where applicable to platform, name of pipeline configuration language	++
Wratten *et al.* [38]	Individual WMSs given 1–3 filled circles, with half-circles allowed	●●○

Descriptions of the scoring systems used in the included papers. Papers that did not assign scores are not included. Examples are given of how scores were represented.

In addition to representing their scores in different ways, the included papers also varied in how clearly they set out their method for assigning these scores. Nine papers did not provide any explanation for how their scores were decided [20, 23, 26, 29, 31, 32, 34–36]. Seven papers provided some context, either within their criteria definitions, elsewhere in the text, or, in the case of Beukers and Allmer in the text summaries provided to justify each score [16, 21, 22, 24, 27, 28, 33]. Most of these papers used a two-level or Yes/No scoring system, which didn’t require an explanation for straightforward criteria such as the presence of a feature. The meaning of the scores was less clear for the more subjective criteria or when additional levels were used. For example, Shi and Wang scored ‘Ease of development’ using three levels, without specifying what was required to achieve each level [35]. Similarly, neither Marinova and Lazarov nor the Leipzig paper they derived their scores from gave any explanation beyond the criteria definitions for how platforms were assigned their five different scoring levels [1, 33].

Two papers not only defined their criteria but also set out exactly what was required to achieve specific scores in each category. Wratten *et al.* defined three levels for each criterion [38]. For example, ease of use was scored as three if the WMS provided a GUI with the execution environment, two if there was a programming interface with an execution environment, or one if the development and execution environments were separate. Kiran *et al.* also defined up to four levels for their criteria, based on specific features or abilities that WMSs can provide [30]. For example, for reusability, level one was assigned if it was possible to use a workflow to analyse different data of the same type with the same intention, level two if workflows could be used to analyse somewhat different data with different intentions, and level three if it was possible to use parts of a workflow to build new workflows.

The variety of these scoring systems is apparent in Table 4, which shows the scores assigned to three WMSs for one similar criterion across studies. The differences are more than aesthetic, since the definitions of the criteria (discussed above), the number of levels used, and the method for assigning the scores vary dramatically. The results from these three studies are not directly comparable, so it would not be possible to combine the results from Karim *et al.* with those of Marinova and Lazarov to compare KNIME and Nextflow [28, 33]. Given the limited information on how these scores were assigned, it would also be impossible to fill in these gaps by reproducing their methods. While Wratten *et al.* provided enough information to allow their method to be reproduced on additional WMSs or the updated versions of these platforms, their criteria only considered certain aspects of the user experience, including this evaluation of ease of use based solely on interface type [38]. Welivita *et al.* implemented the reusable System Usability Scale in addition to their own evaluations, but this is beyond the scope of this review and it would be too time-consuming for most users to repeat when selecting a platform [37].

**Table 4:** Scoring usability.

**Paper and criterion**	**Criteria**	**KNIME**	**Nextflow**	**Galaxy**	**Snakemake**
Karim *et al.* [28]	Ease of use	1	NA	1	NA
Marinova and Lazarov [33]	Ease of use (evaluated within groups)	NA	★★★★★	★★★★	★★★★★
Wratten *et al.* [38]	Ease of use	●●●	●●○	●●●	●●○

Scores assigned to four commonly reviewed WMSs for ‘ease of use’ are presented as in the original papers. NA = platform was not evaluated in that paper. Marinova and Lazarov [33] evaluated groups of similar WMSs, with Nextflow and Snakemake in the same group.

## Discussion

A scoping review was performed to identify the WMS characteristics that improve user experience and explore how these have previously been evaluated in order to understand the factors users should consider when choosing a platform. The review identified 21 relevant papers from a 7-year period, with only four papers concentrating on the needs of noncomputational users and one considering them alongside computational users (Table 1, Fig. 2). While the search strategy did not attempt to identify all WMSs, 55 currently available platforms were identified, providing an overview of available platforms and the characteristics they offer to users (Supplementary Table S2). The variety of these WMSs, which included free and commercial options, online and installable platforms, code-based systems, and GUIs, reveals why comparing platforms is so difficult, but also highlights the dramatic impact platform choice can have on users.

The diversity of the platforms being compared and the users whose needs were being evaluated helps explain why the criteria used to evaluate WMSs varied so much. However, it leaves users unable to draw meaningful comparisons between WMSs based on evaluations in different papers. Even when the same platforms were evaluated on similar-sounding criteria, differences in how these criteria were defined and scored make the results incomparable (Table 4). A full systematic review or meta-analysis would therefore be impossible. Since details of how criteria were defined and scored weren’t always provided, users would also be unable to repeat the evaluations to compare additional platforms or update the scores from older papers.

Twelve shared themes representing the characteristics that benefit users and enable them to tackle common issues in bioinformatics emerged from the criteria used to evaluate WMSs: Basic Computing, Functions, Security, Scalability, Cost/Efficiency, Sustainability, Usability, Learnability, Reproducibility, FAIRness, Flexibility, and Support. These broad themes were consistently present across evaluation papers aimed at different types of users and in the material produced by the WMS developers, indicating they are common concerns. All WMS users want a reliable, usable platform that enhances reproducibility, enables FAIRness, and provides the functions they need.

Although broadly similar themes appeared across sources, differences were apparent in which aspects of these WMS characteristics were valued. Papers focusing on developers and computational users prioritized complex workflow functions, text or code-based interfaces, and the flexibility to bring in new tools and express new ideas. In contrast, papers considering the needs of users with little or no computational experience favoured platforms with pre-installed tools, ready to run pipelines, and GUIs. Similarly, developers of these GUI-based WMSs were more likely to highlight the availability of learning material and support services, emphasizing their importance for the noncomputational users these interfaces are likely to attract, even though these themes were rarely evaluated in review papers.

The papers included in this review were limited to those published since 2018, which made the search process more manageable but also excluded earlier papers such as Leipzig [1]. However, since these older papers often described platforms like Taverna that are no longer maintained, this ensured the extracted criteria were relevant to currently available WMSs. The search terms were designed to identify papers that focused on evaluating WMSs, so some papers that included relevant evaluations as a secondary element may also have been missed. However, including these papers would have dramatically increased the time needed to complete the selection process.

Despite these limitations, this review has highlighted the characteristics of WMSs that matter to users by identifying common themes across previous reviews and materials published by platform developers. By exploring how these characteristics have previously been evaluated, it has also revealed how difficult it can be for life scientists to find the best WMS for their needs. Users cannot simply choose the highest-scoring WMS from a review paper, without checking exactly how the criteria were defined and scored, or considering whose needs were being prioritized by the authors. A WMS that scored highly in a paper based on developer needs won’t necessarily be the best choice for a user who wants to run analyses rather than build tools. However, with few papers focusing on the needs of life scientists, these users have limited information and no clear, reproducible approach for comparing and choosing the most suitable WMS.

In the absence of such a framework, life scientists should treat comparison papers with caution when selecting a WMS. Reported scores should be interpreted in the context of when the evaluations were performed, how criteria were defined and scored, and whether the evaluations reflect the needs of comparable user groups. Users should also be aware of the inherent trade-offs between graphical and code-based interfaces, and between platforms that score highly for criteria relating to the opposing themes of Support and Flexibility.

The issues identified in previous evaluations also suggest four broad principles that should be considered when designing a WMS evaluation framework.

Principle 1: Criteria should be explicitly defined, with sufficient detail to enable interpretation and comparison across studies.

Principle 2: Scoring systems should be described in enough detail to enable interpretation, reproduction of scores, or reuse on new or updated platforms.

Principle 3: Evaluations should clearly state the intended user group or context for which platforms are being assessed.

Principle 4: Trade-offs between competing WMS characteristics should be made explicit and not concealed within aggregate scores.

These principles provide a basis for improving the transparency, reproducibility, and interpretability of WMS evaluations. Future work will build on these principles and the findings of this review to develop and apply a structured evaluation framework for bioinformatics WMSs based on the needs of users.

## Conclusion

The 12 themes identified in this scoping review represent the WMS characteristics that shape the user experience and enable life scientists to address common challenges in bioinformatics. Users should consider these characteristics when choosing a platform, but the variable and sometimes poorly defined criteria used in previous evaluations do not provide a clear approach for comparing WMSs, particularly when the aspects that matter most will depend on the user’s computational background. Users need clearly defined criteria and a reusable evaluation method to help them choose a WMS.

**Key Points**

- Twelve common themes emerge from the user needs and experiences evaluated in previous reviews of WMSs or highlighted by platform developers: Basic Computing, Functions (e.g. tools, workflow structures), Security, Scalability, Cost/Efficiency, Sustainability, Usability, Learnability, Reproducibility, FAIRness, Flexibility, and Support.
- Evaluations focusing on the needs of users with limited programming experience favoured platforms that provided Graphical User Interfaces and plenty of support, while those focused on developers preferred more flexible, text-based platforms.
- Since evaluation criteria weren’t always defined and scoring systems weren’t explained clearly, the results could not be compared between reviews and would be impossible to repeat on new or updated platforms.
- A clear, reusable evaluation framework that considers the user’s requirements and programming experience is needed to enable life scientists to select the most appropriate WMS.

## Supplementary Material

Supplementary_Material_bbag396

Supplementary_Tables_2_3_4_bbag396(1)

## Data Availability

Data supporting this review are included within the article and/or supporting materials.
