# Supplementary material for: A scoping review of approaches to evaluating workflow management systems for bioinformatics users
Source: Brief Bioinform. 2026 Jul 20;27(4):bbag396. doi: 10.1093/bib/bbag396 (PMC13384634; doi:10.1093/bib/bbag396)
Supplement: Supplementary_Material_bbag396 [file supplementary_material_bbag396.docx]

# Supplementary Material

[Supplementary Material 1](#_Toc228996823)

[PRISMA Scoping Review Reporting Checklist 2](#_Toc228996824)

[Supplementary Text 1: Search Strategy 3](#_Toc228996825)

[Supplementary Table 1: Data Items 3](#_Toc228996826)

[Supplementary Figure 1: Subthemes Assigned to Evaluation Criteria 5](#_Toc228996827)

[Supplementary Figure 2: Subthemes Assigned to WMS Characteristics 6](#_Toc228996828)

[Supplementary Table 5a: Themes Identified in Included Papers 1](#_Toc228996829)

[Supplementary Table 5b: Themes Identified in Included Papers by User Focus 2](#_Toc228996830)

[Supplementary Table 6a: Themes Identified in WMS Materials 4](#_Toc228996831)

[Supplementary Table 6b: Themes Identified in WMS Materials by WMS Type 6](#_Toc228996832)

## PRISMA Scoping Review Reporting Checklist

Page numbers are given to identify the section of the review that fulfills each item on the PRISMA checklist **(**[**Scoping — PRISMA statement**](https://www.prisma-statement.org/scoping)**)**

- Title - identifying as a scoping review (p1)

Abstract

- Structured summary (background, objectives, eligibility criteria, sources of evidence, charting methods, results, conclusions relating to the review Qs and objectives) (p2)

Intro

- Rationale (in context of what is already known, why scoping review is appropriate) (p3-6)
- Objectives / Questions (state explicitly, details on populations, contexts etc) (p5)

Methods

- Protocol and Registration (if and where protocol can be accessed) (p6)
- Eligibility Criteria (justify rationale) (p8-9)
- Information Sources (databases with dates of coverage, contact with authors for additional information etc & date of most recent search) (p7-8)
- Search (include full search strategy for at least one database) (p7, Supplementary Text 1)
- Selection of sources of evidence 1 (process for selecting sources) (p8)
- Data charting process (methods e.g. forms that were calibrated or tested before use, was it done independently or in duplicate) (p9-10)
- Data items (list and define all variables and any assumptions or simplifications made) (p10, Supplementary Table 1)
- *Critical appraisal of individual sources of evidence (If done, provide rationale for doing it, method, and how this info was used in any data synthesis) NOT RELEVANT (See p10)*
- Synthesis of results (methods of handling and summarising the data that were charted) (p10-11)

Results

- Selection of sources of evidence (numbers of sources at each stage, with reasons for exclusion at each stage - ideally a flow diagram) (p11, Figure 1)
- Characteristics of sources of evidence (for each source present characteristics for which data were charted, citations) (p12-15, Table 1, Supplementary Table 2)
- *Critical appraisal within sources of evidence (If done, present for each source) NOT RELEVANT*
- Results of individual sources of evidence (for each, present relevant data that were charted that relate to the review Qs/objs) (p12-26, Tables 1 and 3, Supplementary Tables 2, 3, 4, 5 and 6)
- Synthesis of results (summarise the charting results as they relate to the review Qs and objectives) (p12-26, Table 2, Figures 2, 3, 4, 5, and 6)

Discussion

- Summary of evidence (summarise main results, including overview of concepts, themes, types of evidence available, link to the review Qs/objs, consider relevance to key groups) (p27-30)
- Limitations (p29)
- Conclusions (provide general interpretation of the results w.r.t. Review Qs/objs, as well as potential implications/next steps (p31-32)

Funding

- Funding (include sources of funding for the included sources of evidence as well as the review itself) (p32)

## Supplementary Text 1: Search Strategy

The search strategy combined terms relating to bioinformatics (in Titles/Abstracts) AND terms relating to WMS (in Titles) AND terms relating to evaluation/comparison or concepts likely to be evaluated (in Titles).

*The following search terms were used in PubMed and adapted to the other databases:*

(bioinformatics[Title/Abstract] OR biomedical[Title/Abstract]) AND ("Workflow Manage*"[Title] OR system[Title] OR platform[Title] OR toolkit[Title] OR tools[Title] OR workbench[Title] OR engine[Title] OR suite[Title] OR workflow*[Title] OR pipeline*[Title] OR interface[Title] OR framework[Title] OR environment[Title] OR computational[Title] OR desktop[Title] OR cloud[Title] OR GUI[Title] OR app[Title]) AND (benchmark[Title] OR evaluat*[Title] OR compar*[Title] OR review[Title] OR test[Title] OR choose[Title] OR choice[Title] OR select[Title] OR introduce[Title] OR new[Title] OR next[Title] OR generation[Title] OR update*[Title] OR develop*[Title] OR improv[Title] OR best[Title] OR better[Title] OR design[Title] OR accessib*[Title] OR sustainab*[Title] OR reproducible[Title] OR scalab*[Title] OR scaleab*[Title] OR analysis[Title] OR FAIR[Title] OR portab*[Title] OR interoperab*[Title] OR experience[Title] OR use[Title] OR user[Title] OR usability[Title] OR facilitate*[Title] OR community[Title] OR collaborat*[Title] OR community[Title])

## Supplementary Table 1: Data Items

*Fields included in the extraction forms for WMS reviews or platform papers and websites. Both extraction forms also included sections for suggesting additional items to extract (which could then be added to the extraction forms).*

| **Papers or Reviews** | **Platform Papers or Websites** |
| --- | --- |
| - Title - Author (DATE) - Overall aim/ purpose of paper (e.g. comparing reproducibility of WMS, benchmarking WMS) - Scope (e.g. Bioinformatics, Transcriptomics) - Summarise method used - Summarise main findings - List names of criteria used to evaluate WMS - Definitions given of the criteria - Scoring system used (range of scores, any method used to assign scores) - Names of WMS evaluated or mentioned - Scores given for each platform - Target users or audience considered (e.g. Biologists, Developers) - Full citation - Papers identified from Reference/Citation Search for inclusion in this review | - Platform Name - Source Type (PAPER or WEBSITE) - Link to source - Aim/Purpose of Source (e.g. update paper, documentation) - Date platform created - Platform currently available/maintained - Brief summary of platform - Cost (Free/Paid, License type) - How to access (e.g. online, cloud, download) - How to interact (e.g. GUI, command line) - General scope (e.g. Bioinformatics, Genomics) - Characteristics named or highlighted (e.g. as section headings, lists of key features) - Brief summary of the descriptions provided for each characteristic - Full citation |

## Supplementary Figure 1: Subthemes Assigned to Evaluation Criteria

Bars represent the number of criteria assigned to each subtheme. Each theme is presented as a separate panel. Each criterion was assigned to a single subtheme that was the closest match to its name, definition, or description in the paper.


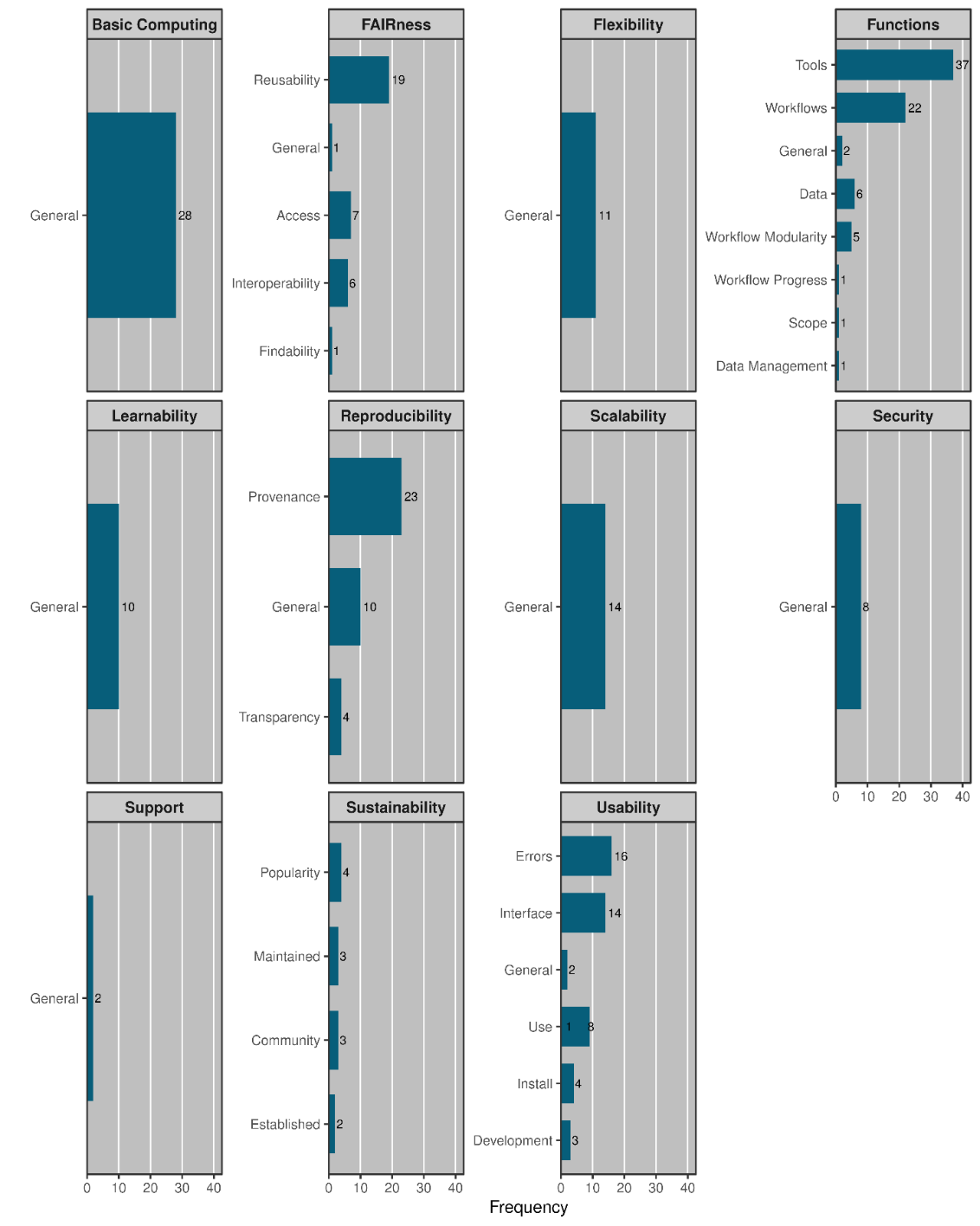


## Supplementary Figure 2: Subthemes Assigned to WMS Characteristics

Bars represent the number of times each subtheme was assigned to the characteristics highlighted in WMS materials. Multiple subthemes were assigned to characteristics that covered multiple areas of user experience or platform use. Each theme is presented as a separate panel.


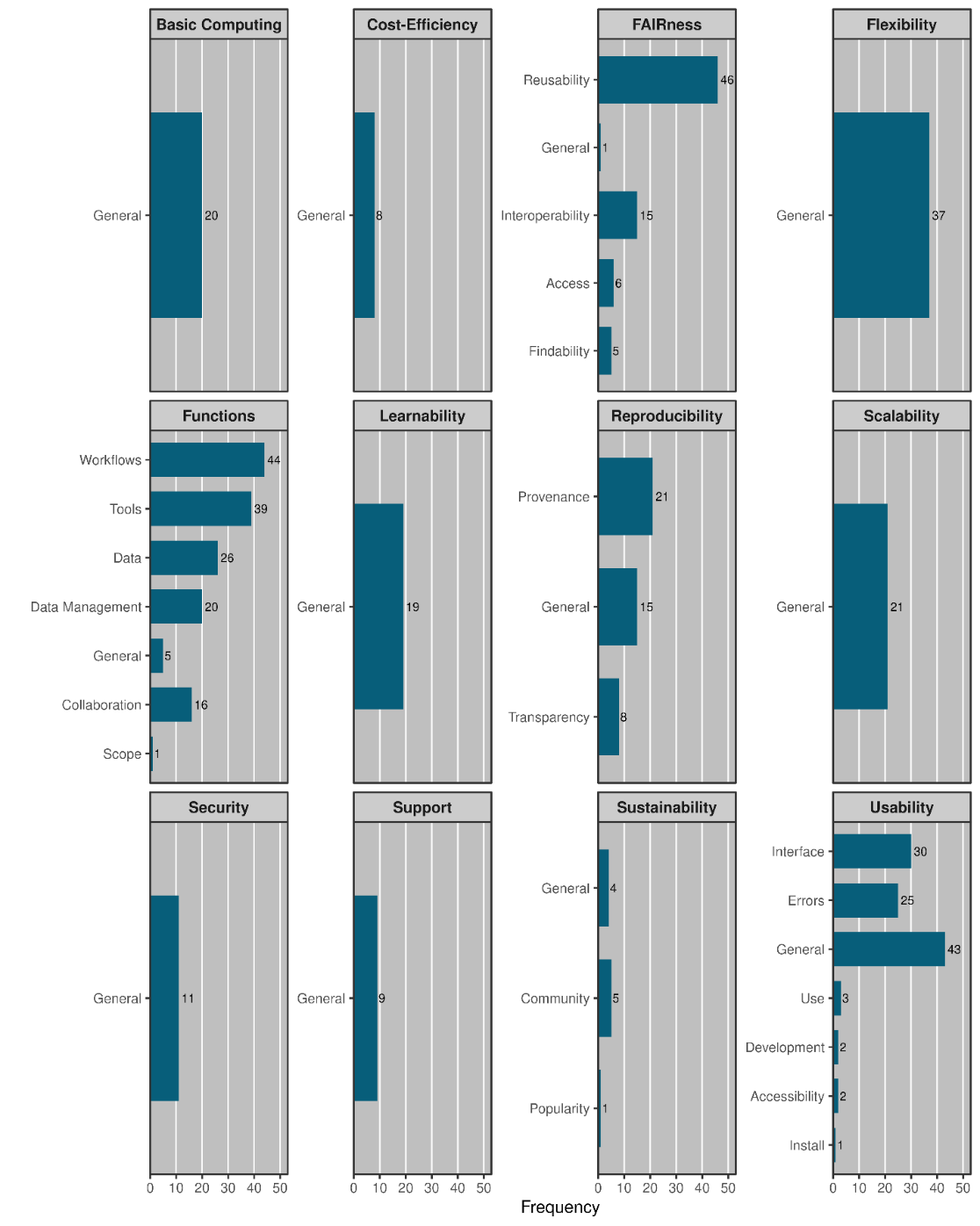


## Supplementary Table 5a: Themes Identified in Included Papers

*Shows the number of criteria from each included paper that were assigned to the themes. Papers marked with * were platform papers that also met the inclusion criteria for evaluation papers.*

| **User Types** | **Paper** | **Basic Computing** | **Functions** | **Security** | **Scalability** | **Sustainability** | **Usability** | **Learnability** | **FAIRness** | **Reproducibility** | **Cost / Efficiency** | **Flexibility** | **Support** | **TOTAL** |
| --- | --- | --- | --- | --- | --- | --- | --- | --- | --- | --- | --- | --- | --- | --- |
| **Technical** | *Ahmed et al., 2021* | 0 | 2 | 0 | 1 | 1 | 3 | 0 | 2 | 1 | 0 | 1 | 0 | 11 |
|  | *Larsonneur et al., (2018)* | 0 | 1 | 0 | 0 | 0 | 0 | 0 | 1 | 1 | 0 | 0 | 0 | 3 |
| **Developers** | ** Cickovski and Narasimhan (2018)* | 0 | 0 | 0 | 0 | 0 | 1 | 2 | 0 | 0 | 0 | 0 | 0 | 3 |
|  | *Jackson et al., (2021)* | 1 | 2 | 0 | 0 | 4 | 3 | 1 | 1 | 0 | 0 | 0 | 0 | 12 |
|  | *Shi and Wang (2019)* | 0 | 0 | 0 | 2 | 0 | 2 | 0 | 0 | 0 | 0 | 0 | 0 | 4 |
| **Programming Experience** | ** Cervera et al., (2019)* | 3 | 2 | 1 | 1 | 1 | 3 | 0 | 1 | 0 | 0 | 0 | 0 | 12 |
|  | ** Fowler et al., (2019)* | 0 | 3 | 0 | 0 | 0 | 4 | 0 | 0 | 5 | 0 | 0 | 0 | 12 |
| **Non-computational** | *Beukers and Allmer (2023)* | 3 | 35 | 1 | 0 | 3 | 9 | 0 | 8 | 1 | 0 | 4 | 2 | 66 |
|  | ** Kinjo et al., (2018)* | 0 | 6 | 0 | 0 | 0 | 1 | 0 | 3 | 0 | 0 | 0 | 0 | 10 |
|  | *Kiran et al., (2023)* | 0 | 7 | 1 | 2 | 2 | 3 | 1 | 5 | 3 | 0 | 1 | 0 | 25 |
|  | ** Tranchant-Dubreuil et al. (2018)* | 1 | 1 | 0 | 1 | 1 | 6 | 1 | 0 | 3 | 0 | 0 | 0 | 14 |
| **Both** | *Wratten et al., (2021)* | 0 | 0 | 0 | 1 | 0 | 1 | 1 | 2 | 0 | 0 | 1 | 0 | 6 |
| **User Type Not Specified** | ** An et al., (2020)* | 1 | 3 | 1 | 0 | 0 | 0 | 0 | 2 | 1 | 0 | 0 | 0 | 8 |
|  | *Boujdad et al., (2019)* | 3 | 0 | 3 | 3 | 0 | 0 | 0 | 0 | 0 | 0 | 0 | 0 | 9 |
|  | *Djaffardjy et al., (2023)* | 0 | 1 | 0 | 0 | 0 | 0 | 0 | 2 | 1 | 0 | 0 | 0 | 4 |
|  | ** Kämpf et al., (2019)* | 9 | 1 | 0 | 0 | 0 | 2 | 0 | 1 | 12 | 0 | 1 | 0 | 26 |
|  | *Karim et al., (2018)* | 2 | 6 | 0 | 2 | 0 | 1 | 1 | 4 | 2 | 0 | 2 | 0 | 20 |
|  | ** Kluge et al., (2020)* | 1 | 3 | 0 | 0 | 0 | 2 | 2 | 2 | 4 | 0 | 1 | 0 | 15 |
|  | *Marinova and Lazarov (2019)* | 0 | 0 | 0 | 1 | 0 | 2 | 0 | 0 | 0 | 0 | 0 | 0 | 3 |
|  | ** Rowe et al., (2019)* | 3 | 1 | 0 | 0 | 0 | 4 | 1 | 0 | 1 | 0 | 0 | 0 | 10 |
|  | ** Welivita et al., (2018)* | 1 | 1 | 1 | 0 | 0 | 1 | 0 | 0 | 2 | 0 | 0 | 0 | 6 |
| *TOTAL* | | 28 | 75 | 8 | 14 | 12 | 48 | 10 | 34 | 37 | 0 | 11 | 2 | 279 |

## Supplementary Table 5b: Themes Identified in Included Papers by User Focus

*Summarises the number of criteria assigned to the themes by papers considering the needs of different user types.*

| **User Types** | **Number of Papers** | **Basic Computing** | **Functions** | **Security** | **Scalability** | **Sustainability** | **Usability** | **Learnability** | **FAIRness** | **Reproducibility** | **Cost / Efficiency** | **Flexibility** | **Support** | **TOTAL** |
| --- | --- | --- | --- | --- | --- | --- | --- | --- | --- | --- | --- | --- | --- | --- |
| **Technical** | 2 | 0 | 3 | 0 | 1 | 1 | 3 | 0 | 3 | 2 | 0 | 1 | 0 | 14 |
| **Developers** | 3 | 1 | 2 | 0 | 2 | 4 | 6 | 3 | 1 | 0 | 0 | 0 | 0 | 19 |
| **Programming Experience** | 2 | 3 | 5 | 1 | 1 | 1 | 7 | 0 | 1 | 5 | 0 | 0 | 0 | 24 |
| **Non-computational** | 4 | 4 | 49 | 2 | 3 | 6 | 19 | 2 | 16 | 7 | 0 | 5 | 2 | 115 |
| **Both** | 1 | 0 | 0 | 0 | 1 | 0 | 1 | 1 | 2 | 0 | 0 | 1 | 0 | 6 |
| **User Type Not Specified** | 9 | 20 | 16 | 5 | 6 | 0 | 12 | 4 | 11 | 23 | 0 | 4 | 0 | 101 |
| TOTAL | - | 28 | 75 | 8 | 14 | 12 | 48 | 10 | 34 | 37 | 0 | 11 | 2 | 279 |

## Supplementary Table 6a: Themes Identified in WMS Materials

*Shows the number of times a theme was identified in the characteristics highlighted by the developers of each platform.*

| **Cost** | **Interface** | Platform | Basic Computing | Functions | Security | Scalability | Sustainability | Usability | Learnability | FAIRness | Reproducibility | Cost / Efficiency | Flexibility | Support | **TOTAL** |
| --- | --- | --- | --- | --- | --- | --- | --- | --- | --- | --- | --- | --- | --- | --- | --- |
| **Free** | **Code/Text** | *Anduril* | 0 | 1 | 0 | 0 | 0 | 1 | 1 | 0 | 1 | 0 | 1 | 0 | 5 |
|  |  | *Apache Airflow (+ Spark)* | 1 | 0 | 0 | 1 | 1 | 4 | 0 | 1 | 0 | 0 | 3 | 0 | 11 |
|  |  | *Arvados* | 1 | 4 | 2 | 1 | 0 | 1 | 0 | 0 | 2 | 0 | 0 | 0 | 11 |
|  |  | *BioLegato* | 0 | 1 | 0 | 0 | 0 | 2 | 0 | 0 | 0 | 1 | 3 | 0 | 7 |
|  |  | *bpipe* | 1 | 1 | 0 | 0 | 0 | 3 | 0 | 0 | 2 | 0 | 0 | 0 | 7 |
|  |  | *Compi* | 0 | 1 | 0 | 0 | 0 | 2 | 0 | 3 | 0 | 0 | 2 | 0 | 8 |
|  |  | *Cosmos* | 1 | 5 | 0 | 0 | 0 | 3 | 1 | 0 | 2 | 0 | 1 | 0 | 13 |
|  |  | *Cromwell* | 0 | 0 | 0 | 0 | 0 | 0 | 0 | 0 | 0 | 0 | 0 | 0 | 0 |
|  |  | *cwltool* | 0 | 0 | 0 | 0 | 0 | 0 | 0 | 0 | 0 | 0 | 0 | 0 | 0 |
|  |  | *Eoulsan* | 1 | 0 | 0 | 0 | 0 | 1 | 0 | 1 | 1 | 0 | 1 | 0 | 5 |
|  |  | *GenPipes* | 1 | 7 | 0 | 1 | 0 | 3 | 0 | 3 | 0 | 0 | 1 | 0 | 16 |
|  |  | *Gwf* | 0 | 0 | 0 | 0 | 0 | 0 | 0 | 0 | 0 | 0 | 1 | 0 | 1 |
|  |  | *JUDI* | 0 | 1 | 0 | 0 | 0 | 2 | 0 | 2 | 0 | 0 | 0 | 0 | 5 |
|  |  | *Luigi* | 0 | 0 | 0 | 0 | 0 | 0 | 0 | 0 | 0 | 0 | 0 | 0 | 0 |
|  |  | *Nextflow* | 2 | 4 | 0 | 1 | 0 | 4 | 0 | 4 | 2 | 0 | 1 | 0 | 18 |
|  |  | *NGOMICS-WF* | 0 | 0 | 0 | 1 | 0 | 2 | 0 | 0 | 0 | 0 | 1 | 0 | 4 |
|  |  | *NGSANE* | 1 | 5 | 1 | 0 | 0 | 2 | 0 | 4 | 5 | 0 | 1 | 1 | 20 |
|  |  | *Pachyderm* | 1 | 4 | 0 | 1 | 0 | 0 | 0 | 1 | 2 | 0 | 0 | 0 | 9 |
|  |  | *Pegasus* | 2 | 5 | 0 | 1 | 0 | 4 | 0 | 1 | 1 | 0 | 0 | 0 | 14 |
|  |  | *Ruffus* | 0 | 1 | 0 | 1 | 0 | 5 | 0 | 0 | 2 | 0 | 0 | 0 | 9 |
|  |  | *SciPipe* | 0 | 3 | 0 | 0 | 0 | 2 | 0 | 2 | 1 | 0 | 1 | 0 | 9 |
|  |  | *Snakemake* | 0 | 8 | 0 | 2 | 0 | 4 | 0 | 4 | 5 | 0 | 4 | 0 | 27 |
|  |  | *SyQADA* | 1 | 2 | 0 | 0 | 0 | 4 | 2 | 3 | 1 | 0 | 0 | 0 | 13 |
|  |  | *targets* | 0 | 1 | 0 | 0 | 0 | 1 | 0 | 0 | 0 | 0 | 0 | 0 | 2 |
|  |  | *Tibanna* | 0 | 0 | 0 | 0 | 0 | 0 | 0 | 0 | 0 | 0 | 0 | 0 | 0 |
|  |  | *TOGGLe* | 0 | 1 | 0 | 1 | 0 | 1 | 0 | 0 | 0 | 0 | 0 | 0 | 3 |
|  |  | *UAP* | 0 | 0 | 0 | 0 | 0 | 3 | 0 | 0 | 2 | 0 | 0 | 0 | 5 |
|  | **GUI** | *ASAP* | 0 | 0 | 0 | 1 | 0 | 0 | 0 | 0 | 1 | 0 | 0 | 0 | 2 |
|  |  | *BioQUEUE* | 0 | 3 | 0 | 0 | 0 | 1 | 2 | 3 | 0 | 0 | 0 | 0 | 9 |
|  |  | *Chipster* | 0 | 4 | 0 | 0 | 0 | 1 | 0 | 1 | 0 | 0 | 1 | 0 | 7 |
|  |  | *Closha/Bio-Express* | 1 | 2 | 0 | 1 | 0 | 4 | 1 | 2 | 0 | 0 | 1 | 0 | 12 |
|  |  | *CyVerse* | 0 | 5 | 3 | 0 | 4 | 2 | 2 | 2 | 0 | 0 | 1 | 0 | 19 |
|  |  | *Galaxy* | 1 | 6 | 0 | 0 | 2 | 9 | 2 | 5 | 0 | 0 | 1 | 0 | 26 |
|  |  | *GenePattern* | 0 | 0 | 0 | 0 | 0 | 2 | 0 | 1 | 2 | 0 | 0 | 0 | 5 |
|  |  | *Kepler* | 0 | 9 | 0 | 0 | 1 | 1 | 0 | 3 | 0 | 0 | 0 | 0 | 14 |
|  |  | *KNIME* | 0 | 11 | 0 | 1 | 2 | 2 | 1 | 5 | 1 | 1 | 4 | 2 | 30 |
|  |  | *Maser* | 0 | 3 | 0 | 0 | 0 | 3 | 1 | 5 | 3 | 0 | 1 | 1 | 17 |
|  |  | *NEAT* | 0 | 1 | 0 | 0 | 0 | 2 | 0 | 0 | 1 | 0 | 0 | 0 | 4 |
|  |  | *OpenBio-C* | 0 | 9 | 0 | 1 | 0 | 1 | 1 | 2 | 1 | 0 | 1 | 1 | 17 |
|  |  | *UniPro UGENE* | 0 | 4 | 0 | 0 | 0 | 3 | 0 | 1 | 0 | 0 | 0 | 0 | 8 |
|  |  | *Watchdog* | 1 | 1 | 0 | 1 | 0 | 5 | 0 | 0 | 0 | 0 | 2 | 0 | 10 |
|  |  | *Wings* | 2 | 3 | 0 | 0 | 0 | 1 | 0 | 3 | 0 | 0 | 0 | 0 | 9 |
| **Paid** |  | *BioData Catalyst* | 0 | 0 | 0 | 0 | 0 | 0 | 0 | 0 | 0 | 0 | 0 | 0 | 0 |
|  |  | *CAVATICA* | 0 | 3 | 1 | 0 | 0 | 0 | 1 | 1 | 0 | 0 | 0 | 0 | 6 |
|  |  | *CLC Genomics Workbench* | 1 | 1 | 0 | 1 | 0 | 1 | 0 | 1 | 0 | 0 | 0 | 0 | 5 |
|  |  | *DataJoint* | 0 | 1 | 0 | 0 | 0 | 1 | 0 | 1 | 1 | 0 | 0 | 0 | 4 |
|  |  | *DNAnexus* | 0 | 2 | 1 | 0 | 0 | 2 | 0 | 0 | 0 | 2 | 1 | 1 | 9 |
|  |  | *FlowJo / SeqGeq* | 0 | 2 | 0 | 0 | 0 | 1 | 0 | 0 | 0 | 1 | 0 | 1 | 5 |
|  |  | *Geneious* | 0 | 3 | 0 | 0 | 0 | 3 | 1 | 1 | 0 | 0 | 1 | 1 | 10 |
|  |  | *Pipeline Pilot* | 0 | 2 | 0 | 0 | 0 | 2 | 0 | 2 | 0 | 0 | 0 | 0 | 6 |
|  |  | *RapidMiner* | 0 | 9 | 0 | 1 | 0 | 1 | 0 | 0 | 1 | 0 | 0 | 0 | 12 |
|  |  | *SevenBridges (now Velsera)* | 0 | 0 | 1 | 0 | 0 | 1 | 0 | 0 | 1 | 1 | 1 | 0 | 5 |
|  |  | *Shivom* | 0 | 4 | 0 | 0 | 0 | 0 | 0 | 2 | 1 | 2 | 0 | 0 | 9 |
|  |  | *Terra* | 0 | 4 | 2 | 1 | 0 | 0 | 1 | 2 | 1 | 0 | 1 | 0 | 12 |
|  |  | *VarSeq* | 1 | 4 | 0 | 2 | 0 | 3 | 2 | 1 | 1 | 0 | 0 | 1 | 15 |
| *TOTAL* | | | 20 | 151 | 11 | 21 | 10 | 106 | 19 | 73 | 44 | 8 | 37 | 9 | 509 |

## Supplementary Table 6b: Themes Identified in WMS Materials by WMS Type

*Summarises the number of times the themes were identified for different platform types.*

| **Cost** | **Interface** | **Number of Platforms** | **Basic Computing** | **Functions** | **Security** | **Scalability** | **Sustainability** | **Usability** | **Learnability** | **FAIRness** | **Reproducibility** | **Cost / Efficiency** | **Flexibility** | **Support** | **TOTAL** |
| --- | --- | --- | --- | --- | --- | --- | --- | --- | --- | --- | --- | --- | --- | --- | --- |
| **Free** | **Code/Text** | 27 | 13 | 55 | 3 | 11 | 1 | 54 | 4 | 29 | 29 | 1 | 21 | 1 | 222 |
| **Free** | **GUI** | 15 | 5 | 61 | 3 | 5 | 9 | 37 | 10 | 33 | 9 | 1 | 12 | 4 | 189 |
| **Paid** | **GUI** | 13 | 2 | 35 | 5 | 5 | 0 | 15 | 5 | 11 | 6 | 6 | 4 | 4 | 98 |
|  | TOTAL | - | 20 | 151 | 11 | 21 | 10 | 106 | 19 | 73 | 44 | 8 | 37 | 9 | 509 |
